# Triptolide Induces Apoptosis Through Fas Death and Mitochondrial Pathways in HepaRG Cell Line

**DOI:** 10.3389/fphar.2018.00813

**Published:** 2018-07-26

**Authors:** Longtai You, Xiaoxv Dong, Boran Ni, Jing Fu, Chunjing Yang, Xingbin Yin, Xin Leng, Jian Ni

**Affiliations:** ^1^School of Chinese Materia Medica, Beijing University of Chinese Medicine, Beijing, China; ^2^School of Basic Medical Science, Beijing University of Chinese Medicine, Beijing, China; ^3^Beijing Hospital of Traditional Chinese Medicine Affiliated to Capital University of Medicine Sciences, Beijing, China; ^4^Beijing Research Institute of Chinese Medicine, Beijing University of Chinese Medicine, Beijing, China

**Keywords:** triptolide, hepatotoxicity, HepaRG cells, ROS, apoptosis

## Abstract

Triptolide isolated from the traditional Chinese herb *Tripterygium wilfordii* Hook F., possesses anti-tumor, anti-fertility, and anti-inflammatory properties. Triptolide-induced hepatotoxicity has continued to engage the attention of researchers. However, not much is yet known about the cytotoxicity of triptolide, and the precise mechanisms involved. In the present study, we investigated the cytotoxicity of triptolide and its underlying mechanisms, using the *in vitro* model (HepaRG cell). The results demonstrated that triptolide significantly reduced cell viability and induced apoptosis in HepaRG cells in a dose- and time-dependent manner. Triptolide treatment also provoked reactive oxygen species (ROS) generation and depolarization of mitochondrial membrane potential (MMP). Moreover, triptolide dose-dependently increased the protein expression levels of Fas, Bax, p53, p21, cyclin E, cleaved caspase-3, 8, and 9; and subsequent cleavage of poly (ADP-ribose) polymerase (PARP). However, the protein expression of Bcl-2, cyclin A, and CDK 2 were significantly decreased. These results suggest that triptolide inhibits cell proliferation and induces apoptosis via the Fas death pathway and the mitochondrial pathway.

## Introduction

**FIGURE 1:**
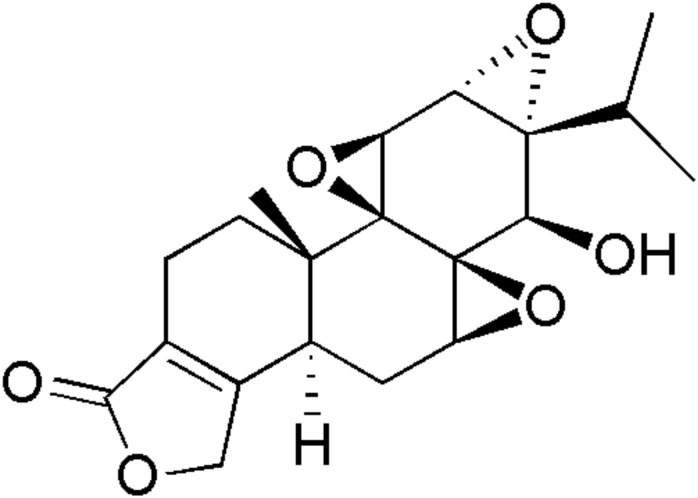
Chemical structure of triptolide.

Triptolide (**Figure 1**) is a diterpenoid triepoxide derived from the root extracts of *Tripterygium wilfordii* Hook F (TWHF) (Du et al., 2014). It possesses a wide range of biological and pharmacological activities, such as anti-tumor, anti-fertility, anti-inflammatory, and immunosuppressive properties (Wang et al., 2013, 2014a). Recent studies reported that triptolide inhibits the viability of various cells such as L-02, HepG2, HK-2, and H9c2 (Xi et al., 2017). The expressions of cytochrome P450s (CYP450s), kinase B (AKT), Bax, Bcl-2, caspases-3, and members of the mitogen-activated protein kinase (MAPK, JAK/STAT, and PI3K-AKT) family, are regulated in triptolide-induced apoptosis (Mei et al., 2005; Li et al., 2014; Shen et al., 2014; Kong et al., 2015; Lu et al., 2017). Reports from several studies showed that triptolide isolated from TWHF exerted significant cytotoxicity in rat primary hepatocytes and HepG2 cells, indicating that triptolide might be one of the main toxic components of TWHF (Wang et al., 2014b; Jin et al., 2015). Moreover, it has been reported that triptolide exposure could result in injury to various organs, such as liver, kidney, testes, ovary, and heart, not only in experimental animals and, but also in humans (Xi et al., 2017).

Apoptosis, a programmed cell death, plays a critical role in the defense against disease and exogenous stresses. It is genetically controlled and regulated by two major pathways: death receptor-mediated pathway (extrinsic), and mitochondrial-dependent pathway (intrinsic) (Zhou et al., 2010; Chiang et al., 2017; Dong X. et al., 2017; Dong Z. et al., 2017). Caspases, a family of cysteine proteases, are characteristically involved in apoptosis (Wen et al., 2012). The extrinsic pathway is triggered by ligation of death receptors and subsequent caspase-8 activation within a death-inducing signaling complex. In contrast, the intrinsic pathway is initiated by intracellular stress, and subsequently activated by caspase-9. Despite the fact that the two pathways are activated by different stimuli, both will directly activate downstream effector caspase-3 (Tsang and Kwok, 2008; Huang et al., 2011). Moreover, the mitochondrial-dependent apoptosis is regulated by the Bcl-2 family proteins, such as Bax, Bak, and Bcl-2 (DiPaola et al., 2001). Changes in Bax/Bcl-2 ratio result in significant activation of caspases, and lead to programmed cell death through the mitochondrial-dependent pathway (Kang and Reynolds, 2009).

In the present study, we investigated the cytotoxic effect of triptolide in HepaRG cells, and the underlying molecular mechanisms. The results demonstrate that triptolide induced cell cycle arrest at G2/M phase and caspase-dependent apoptosis via the Fas death pathway and the mitochondrial pathway through the generation of reactive oxygen species (ROS).

## Materials and Methods

### Reagents

Triptolide(batch no. 2,826, purity >98.0%)was purchased from Shanghai Standard Biotech Co., Ltd. (Shanghai, China). Triptolide solution (16 mM) was prepared in dimethyl sulfoxide (DMSO) and kept at 4°C. The working solution was prepared by dilution of the stock solution in the basal medium before each experiment. The final working concentration of DMSO in experimental conditions was not allowed to exceed 0.1%. Previous studies have shown that the *in vitro* cytotoxicity of triptolide ranges from 5 to 640 nM (Xi et al., 2017). In addition, our previous preliminary experiments showed that triptolide (100–400 nM) inhibited HepaRG cell viability in a concentration-dependent manner. Therefore, we chose triptolide concentration range of 100–400 nM for use in the present study.

Fetal bovine serum (FBS), trypsin and penicillin/streptomycin solution were obtained from Corning (NY, United States), while RPMI 1,640 medium, PBS and MTT were products of Solarbio (Beijing, China). Assay kits for LDH, DAPI, Annexin V-FITC Apoptosis, ROS, MMP and Cell Cycle were supplied by Beyotime (Nanjing, China). Antibodies for Fas (#4233), Bax (#5023T), Bcl-2 (#15071), p53 (#2524T), p21(#2947T), cyclin A(#4656T), CDK 2 (#2546T), cleaved caspase-3 (#9661T), cleaved caspase-9 (#9501T), cytochrome c (#4280T), and PARP (#9542T) were purchased from Cell Signaling Technology, while antibody for caspase-8 (#ab25901) was obtained from Abcam (#ab25901).

### Cell Cultures and Treatment

HepaRG cell line was purchased from Shanghai Guan&Dao Biological Engineering Co., Ltd. (Shanghai, China). The cells were cultured in RPMI 1,640 medium supplemented with 10% FBS, antibiotics (100 U/mL penicillin and 100 μg/mL streptomycin), and incubated at 37°C in a humidified atmosphere containing 5% CO_2_. Trypsin (0.25%, Sigma) was used to passage the cells at 80–90% confluence.

### Cell Viability Assay

In order to evaluate the effect of triptolide on the growth of HepaRG cells, cell proliferation was assessed using a MTT assay. HepaRG cells (5.0 × 10^3^ cells/well) were plated into 96-well plates overnight. The cells were treated with different concentrations of triptolide (0, 100, 200, and 400 nM) for 24, 48, and 72 h, while the control received 0.1% DMSO in place of triptolide. The cells were incubated with new culture medium containing MTT working solution (0.5 mg/mL) for 4 h at 37°C. Thereafter, the culture supernatant was removed from all the wells, and the water-insoluble formazan crystals were dissolved in 150 μL DMSO. The absorbance of the formazan solution was measured at 570 nm in a microplate reader (Thermo, Multiskan, GO, United States).

In the assay of lactate dehydrogenase (LDH), the cells (5.0 × 10^3^ cells/well) were seeded into 96-well plates overnight and then treated with serial concentrations of triptolide for 24 h. The supernatant was used for the assay of LDH activity with a commercial LDH kit according to the manufacturer’s instructions. Absorbance was measured at 490 nm in a microplate reader (Thermo, Multiskan, GO, United States). Dual-wavelength measurements were performed with 600 nm as the reference wavelength. All the experiments were performed in triplicate.

### DAPI Staining

The use of the fluorescent dye DAPI stain for DNA can aid in visual analysis of nuclear morphological changes (Ferro et al., 2017). HepaRG cells (4.0 × 10^5^ cells/well) were seeded and treated with different concentrations of triptolide (0, 100, 200, and 400 nM) for 24 h. The cells were collected and washed once with phosphate-buffered saline (PBS) and fixed with 4% paraformaldehyde for 20 min at room temperature. They were then stained in the dark with DAPI solution (1 μg/ml) for 10 min at room temperature. The cells were washed twice with PBS, and nuclear changes were visualized under an inverted Olympus IX71 fluorescence microscope (Japan) excited at a wavelength of 364 nm and measured at 454 nm.

### Annexin V-FITC and PI Double Staining

The apoptotic cells were detected using an Annexin V-FITC Detection Kit and measured by flow cytometry (Song et al., 2017). HepaRG cells (4.0 × 10^5^ cells/well) were plated in six-well plates and incubated with different concentrations of triptolide (0, 100, 200, and 400 nM) for 24 h at 37°C. The cells were thereafter collected and washed with PBS. Then, they were re-suspended in 295 μl binding buffer and incubated with 5 μl Annexin V-FITC and 10 μl propidiumiodide at 37°C for 20 min in the dark. Finally, the samples were analyzed using flow cytometry (BD FACSCanto II, United States). The fluorescence was measured in fluorescence channels FL1 (488 nm excitation and 530/30 nm emission for FITC-labeled annexin-V) and FL3 (488 nm excitation and 585/42 nm emission for PI).

### Measurement of Intracellular ROS

Intracellular ROS generation was measured using the DCFH-DA fluorescent dye (Du et al., 2017; Wang et al., 2017). The DCFH-DA probe is a non-polar compound which lightly diffuses into cells. Moreover, it is hydrolyzed by intracellular esterase to produce DCFH, which is trapped by the cells. The ROS (hydrogen peroxide, peroxynitrite, or hydroxyl radical) produced by cells oxidize DCFH to form the highly fluorescent compound 2, 7-dichlorofluorescein (DCF) (Eruslanov and Kusmartsev, 2010). In this assay, HepaRG cells were seeded in six-well culture plates at a density of 4 × 10^5^ cells per well and then treated with various concentrations of triptolide (0, 100, 200, and 400 nM) for 24 h. Subsequently, the cells were treatedwith 10 μM DCFH-DA and incubated for 30 min at 37°C in the dark. The cells were harvested, washed twice with PBS and re-suspended for analysis. Finally, fluorescence was measured by flow cytometry at excitation and emission settings of 488 and 530/30 nm, respectively.

### Measurement of Mitochondrial Membrane Potential

Mitochondrial membrane potential (MMP) was measured using JC-1, which is a mitochondria-specific lipophilic cationic fluorescence dye able to selectively enter the mitochondria (Zhang and Zhang, 2017). The HepaRG cells (4.0 × 10^5^ cells/well) were plated in 6-well culture plates and treated with different concentrations of triptolide (0, 100, 200, and 400 nM). They were then further incubated for 24 h. Then, the cells were stained with 10 μM JC-1 working solution for 30 min at 37°C in the dark, washed twice and re-suspended with PBS. The changes in MMP were measured and analyzed with a flow cytometer. The fluorescence was excited at a wavelength of 488 nm and measured at 530/30 nm (green fluorescence) and 585/42 nm (red fluorescence).

### Cell Cycle Analysis

In order to assess the effects of triptolide on cell cycle distribution, the DNA contents of the cells were measured by flow cytometry (Gao et al., 2017). In this assay, HepaRG cells were seeded in six-well plates at a density of 4 × 10^5^ cells/well overnight and treated with different concentrations of triptolide (0, 100, 200, and 400 nM) for 24 h. The cells were harvested, fixed with 70% ice-cold ethanol at 4°C for at least 24 h, and stained in the dark with PI/RNase A staining buffer solution for 30 min at 37°C. The resulting suspension was passed through a nylon mesh filter (diameter: 50 μm) and analyzed via flow cytometry. The fluorescence was measured in fluorescence channels FL3 (488 nm excitation and 585/42 nm emission for PI).

### Western Blot Analysis

HepaRG cells (4.0 × 10^5^ cells/well) were seeded in 6-well plates overnight and treated with different concentrations of triptolide (0, 100, and 200 nM) for 24 h. Then, the HepaRG cells were harvested, washed twice with ice-cold PBS, and lysed with RIPA buffer (Dinguo Changsheng Biotechnology, Beijing, China) on ice for 30 min. The lysates were centrifuged at 15,777 × *g* for 10 min at 4°C. Total protein concentration was determined using BCA protein assay kit (Dinguo Changsheng Biotechnology, Beijing, China). In a parallel experiment, the mitochondrial and cytosolic fractions were separated using ProteoExtract^®^ Cytosol/Mitochondria Fractionation Kit (Millipore, Billerica, MA, United States) according to the manufacturer’s instructions. The protein lysates (50–100 μg) were separated on a 10% sodium dodecyl sulfate-polyacrylamide gel electrophoresis (SDS-PAGE) and transferred to polyvinylidene fluoride (PVDF) membrane (Pall, New York, NY, United States). The membranes were blocked with TBST (25 mM Tris, 150 mM NaCl, 0.1% Tween 20, pH 7.4) buffer containing 5% skim milk for 1h, and then incubated overnight at 4°C with specific primary antibodies at the indicated dilutions: Fas (1:1,000), cytochrome c (1:500), Bcl-2 (1:1,000), Bax (1:1,000), cleaved PARP (1:1,000), cleaved caspase 3 (1:1,000), cleaved caspase 8 (1:1,000), cleaved caspase 9 (1:1,000), cyclin A (1:1,000), cyclin E (1:1,000), CDK2 (1:1,000), p53 (1:1,000), p21 (1:1,000), or β-actin (1:5,000) (Wu et al., 2017). Thereafter, the membranes were further incubated with corresponding secondary antibodies (1:5,000 dilution) at room temperature for 1 h. Finally, an intensive ECL detection system (Pierce, Appleton, WI, United States) was used to visualize the target proteins. The experiments were performed at least thrice.

### Statistical Analysis

Each experiment was performed at least three times and data are presented as mean ± standard deviation (SD). The data are analyzed using version 17.0 SPSS software. One-way analysis of variance (ANOVA) and LSD test were used to determine statistical significance of differences. A difference was considered significant when *p* < 0.05.

## Results

### Triptolide Induced Cytotoxicity in HepaRG Cells

**FIGURE 2:**
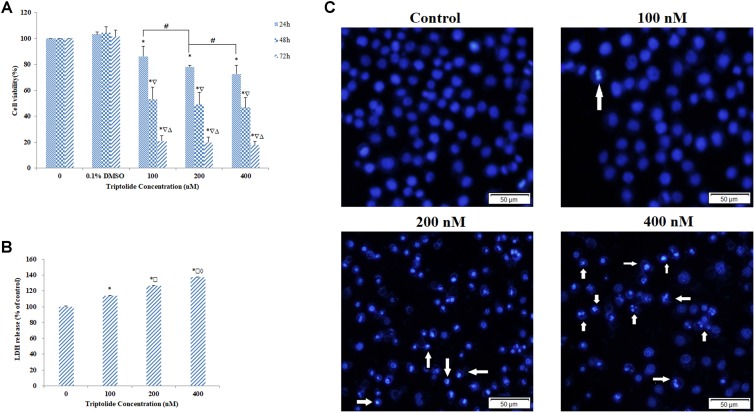
Cell viability and morphology of HepaRG cells treated with various concentrations of triptolide. **(A)** Cell viability as assessed by the MTT assay. **(B)** Cell cytotoxicity evaluated by LDH assay. **(C)** Morphological changes in HepaRG cell nucleus determined by DAPI staining and observed by fluorescence microscopy (original magnification = ×200, Bar = 50 μm). The arrows represent the apoptotic cells. Data are presented as mean ± S.D. of three independent experiments. ^∗^*p* < 0.05, vs. vehicle control; ^
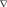
^*p* < 0.05, vs. 24 h-group; ^
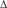
^*p* < 0.05 vs. 48 h-group; ^
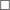
^*p* < 0.05 vs. 100 nM-group; ^
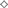
^*p* < 0.05 vs. 200 nM-group; ^#^*p* < 0.05 indicates significant differences between different samples within the same exposure time.

As expected, the results of the MTT assay demonstrated that triptolide inhibited cell growth in a dose- and time-dependent manner, when compared to the vehicle controls (**Figure 2A**). The IC_50_ value of triptolide following 48 h exposure of HepaRG cells was approximately 200 nM. LDH is mainly located in the cytoplasm, and its presence in the extracellular medium is used to detect damage of cell membrane integrity. Therefore, LDH leakage is considered an indication of cell membrane damage. In this study, triptolide treatment led to dose-dependent leakage of LDH from the HepaRG cells (**Figure 2B**). After exposure to triptolide for 24 h, DAPI staining showed clear evidence of apoptosis-related morphological changes, such as condensation of chromatin and nuclear fragmentation (**Figure 2C**).

### Triptolide Induced Apoptosis in HepaRG Cells

**FIGURE 3:**
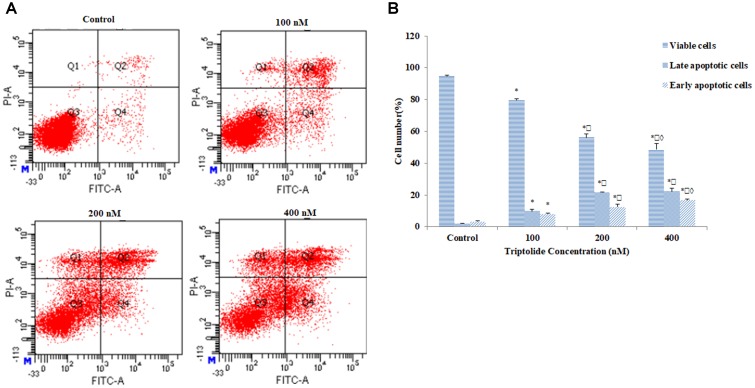
Effect of triptolide on apoptosis in HepaRG cells. **(A)** Apoptosis detection with Annexin V/PI double staining in different groups by flow cytometry. The Q3 region represents viable cells, while Q4 region represents early apoptotic cells. The Q2 region represents late apoptotic cells, while Q1 region represents necrotic cells or mechanical damaged cells. **(B)** Column bar graph of mean cell florescence for viable, early apoptotic and late apoptotic cells. Data are presented as mean ± S.D. of three independent experiments. ^∗^*p* < 0.05, vs. vehicle control; ^
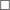
^*p* < 0.05 vs. 100 nM-group; ^
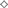
^*p* < 0.05 vs. 200 nM-group.

Results from Anexin V/PI double staining revealed that the percentage of viable cells was significantly lower after treatment with triptolide for 24 h, when compared with the control group. In addition, the percentage of early and late apoptotic cells was significantly increased in a concentration-dependent manner from 3.20% ± 0.52 to 16.77% ± 0.55, and from 1.97% ± 0.32 to 22.40% ± 1.90, respectively (**Figures 3A,B**). Thus, the cell death induced by triptolide in HepaRG cells was due to induction of apoptosis.

### Triptolide Promoted ROS Generation in HepaRG Cells

**FIGURE 4:**
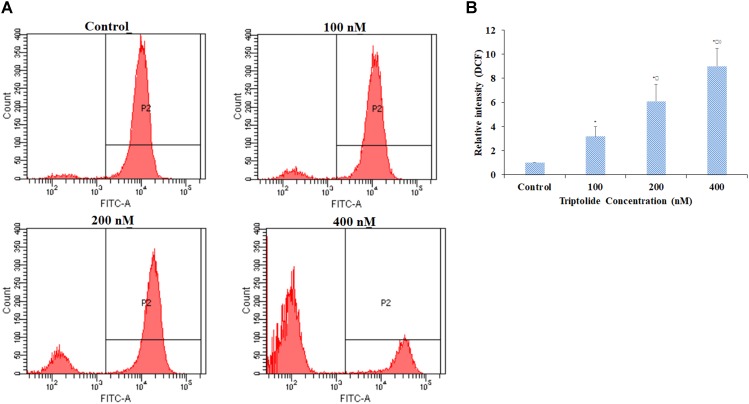
Effect of triptolide on levels of ROS in HepaRG cells. **(A)** ROS detection with DCFH-DA dye in different groups by flow cytometry. **(B)** Column bar graph of mean cell florescence for DCFH-DA. Data are presented as mean ± S.D. of three independent experiments. ^∗^*p* < 0.05, vs. vehicle control; ^
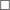
^*p* < 0.05, vs. 100 nM-group; ^
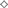
^*p* < 0.05 vs. 200 nM-group.

In order to investigate whether the triptolide-induced apoptosis was associated with ROS generation, the effect of triptolide on ROS levels was determined. Compared with untreated cells, there were significant increases in ROS generation after treatment with various concentrations of triptolide for 24 h (**Figures 4A,B**). Hence, triptolide treatment might impair cellular redox status.

### Triptolide Reduced MMP in HepaRG Cells

**FIGURE 5:**
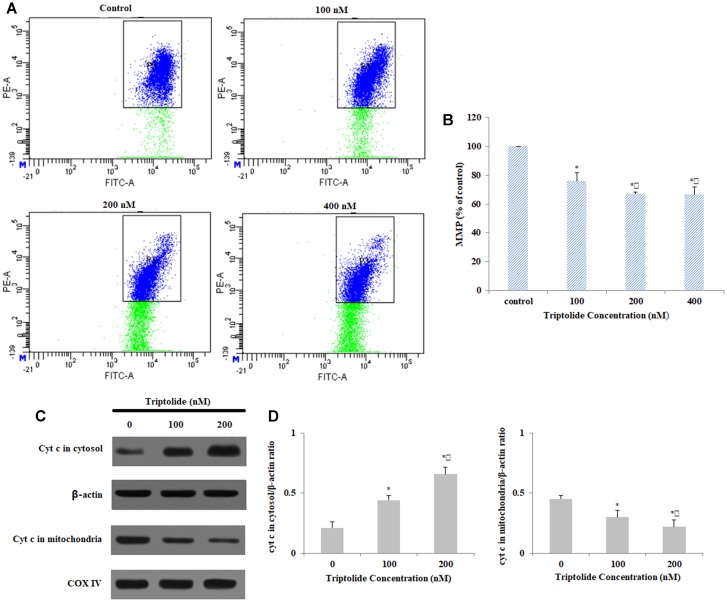
Effect of triptolide on MMP in HepaRG cells. **(A)** MMP detection with JC-1 staining in different groups with flow cytometry. **(B)** Column bar graph of mean cell florescence for JC-1. **(C)** Mitochondrial and cytosolic levels of Cyt c, as determined using Western blotting (β-actin and COX IV were used as the internal control for the cytosolic and mitochondrial fractions, respectively). **(D)** Quantification and analysis of protein-related bands using densitometry. Data are expressed as mean ± S.D. of three independent experiments. ^∗^*p* < 0.05 vs. vehicle control; ^
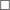
^*p* < 0.05 vs. 100 nM-group.

Previous studies showed that mitochondrial membrane permeabilization could directly cause mitochondrial dysfunction, result in loss of membrane potential, and activate the release of cytochrome c (Yang et al., 2012; Zhang et al., 2017). Concentration-dependent loss of MMP was induced in HepaRG cells by triptolide, relative to the control, untreated group (**Figures 5A,B**). Cytochrome c plays a critical role in the mitochondria-dependent apoptosis pathways. Therefore, we used western blot analysis to determine whether incubation with triptolide enhanced the release of cytochrome c. The results indicated that the triptolide treatment for 24 h led to marked increase in release of cytochrome c from the mitochondria to the cytosol (**Figures 5C,D**). Taken together, these findings demonstrate that mitochondrial dysfunction is implicated in the triptolide-induced apoptosis of HepaRG cells.

### Triptolide Induces G2/M Phase Cell Cycle Arrest in HepaRG Cells

**FIGURE 6:**
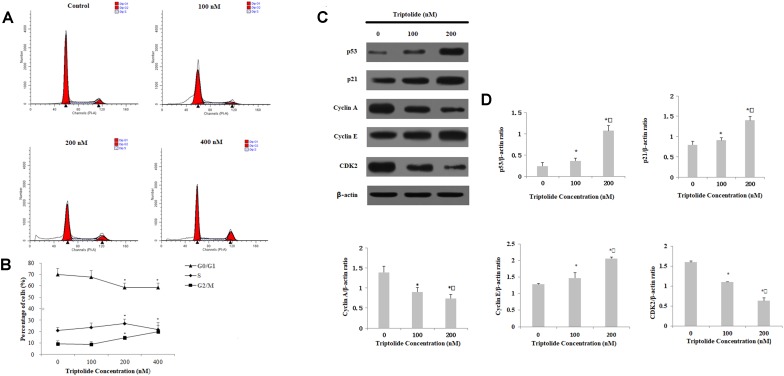
Effect of triptolide on cell cycle distribution in HepaRG cells, measured and analyzed by flow cytometry. **(A)** Triptolide induced cell cycle arrest at the G2/M phase. **(B)** The percentage of HepaRG cells in each phase of the cell cycle treated with different concentrations of triptolide for 24 h. **(C)** Expression levels of relatedproteins measured by Western blotting, with β-actin as a loading control. **(D)** Densitometric and statistical analyses of the protein-related bands. Data image are expressed as mean ± S.D. of three independent experiments. ^∗^*p* < 0.05 vs. vehicle control; ^
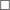
^*p* < 0.05 vs. 100 nM-group.

In order to investigate the effects of triptolide on cell cycle, the HepaRG cells were stained with PI and then analyzed by flow cytometry. After 24 h of triptolide (400 nM) exposure, the percentage of cells in the G2 phase increased significantly from 9.08 ± 2.28% to 19.88 ± 2.18%, while the percentage of cells in the G1 phase decreased from 69.89 ± 5.14% to 58.44 ± 3.67%, when compared with untreated cells (**Figures 6A,B**). To confirm whether triptolide affected changes in the cell cycle, we determined the expression levels of the proteins involved in the G2/M phase progression. The results showed that the expression levels of cyclin E, p53, and p21 proteins were markedly upregulated in a dose-dependent manner by triptolide, while other cyclin proteins (cyclin A and CDK2) were distinctly downregulated (**Figures 6C,D**). This indicates that triptolide can arrest the HepaRG cells at G2/M phase.

### Effects of Triptolide on Levels of Apoptosis-Related Proteins in HepaRG Cells

**FIGURE 7:**
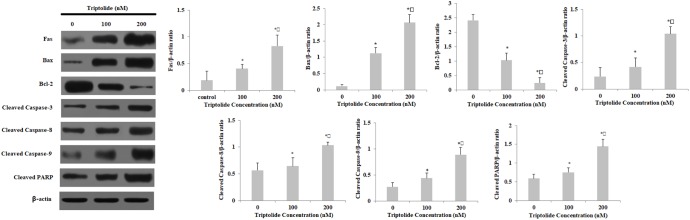
Effect of triptolide on the expression of apoptosis-related proteins in HepaRG cells. Densitometric analysis was used to quantify these protein-related bands and statistically analyze them. Data are expressed as mean ± S.D. of three independent experiments. ^∗^*p* < 0.05 vs. vehicle control; ^
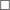
^*p* < 0.05 vs. 100 nM-group.

The expressions of apoptosis-related proteins were measured by western blot in order to further characterize the potential mechanism of triptolide-induced apoptosis (**Figure 7**). The expression of Fas protein, which is a representative death receptor, was increased at 24 h following triptolide treatment. Furthermore, there were significant increases in the expressions of Bax, cleaved caspase-3, 8, and 9; and significant decrease in the expression of Bcl-2. The triptolide treatment also resulted in the cleavage of PARP, which is the substrate of caspase-3. These results suggest that triptolide induces apoptosis in HepaRG cells through the death receptor and the mitochondrial apoptotic pathways.

## Discussion

Triptolide, a diterpenoid triepoxide, has been used clinically to treat inflammatory and autoimmune diseases. Previous studies have shown that triptolide possesses many biological and pharmacological effects (Wang et al., 2013, 2014a; Du et al., 2014). However, results from some studies have suggested that triptolide might be one of the major hepatotoxic components of *Tripterygium wilfordii* Hook F. (Mei et al., 2005; Li et al., 2014; Shen et al., 2014; Wang et al., 2014b; Jin et al., 2015; Kong et al., 2015; Lu et al., 2017; Xi et al., 2017). In the present study, we utilized HepaRG cells to investigate the cytotoxicity of triptolide and the underlying molecular mechanisms.

Results from MTT and LDH assays showed that triptolide significantly reduced HepaRG cell viability in a concentration- and time-dependent manner. Moreover, DAPI and Annexin V/PI double staining showed that triptolide inhibited the growth of HepaRG cells in a dose-dependent manner through apoptosis. Thus, the triptolide-induced cytotoxic effects on HepaRG cells were clearly due to apoptosis.

Excessive ROS generation and/or reduced antioxidant defenses result in lipid peroxidation and, structural changes in relevant proteins, leading to cellular impairment which is a key event in apoptosis (Song et al., 2014). In the present study, triptolide treatment increased the levels of ROS in a dose-dependent manner, suggesting that it induces oxidative stressin HepaRG cells. Moreover, triptolide exposure resulted in the loss of MMP in HepaRG cells and promoted the release of cytochrome c from the mitochondria into the cytosol. These results clearly suggest that triptolide causes mitochondrial damage in the HepaRG cells.

Western blot analysis indicated that triptolide treatment led to significant increases in the expressions of Fas, Bax, p53, p21, and cleaved caspases-3, 8, and 9, PARP, while the expression level of Bcl-2 was markedly down-regulated, relative to the control cells. Furthermore, triptolide enhanced the release of cytochrome c from mitochondria into cytosol. Recent studies have reported that triptolide inhibits the proliferation of HepG2 cells while promoting their apoptosis by increasing the expressions of p53, p21, DR 5, and Bax (Sun et al., 2017). Moreover, it has been shown that triptolide induced apoptosis in human laryngocarcinoma HEp-2 cells and HL-60 cells through the death receptor-mediated pathway and the mitochondrial-dependent pathway (Wan et al., 2006; Zhao et al., 2013). The results obtained in the present study are consistent with these reports. Our results showed that triptolide induced apoptosis in HepaRG cells through the Fas death receptor-mediated and the caspase-dependent mitochondrial apoptotic pathways.

## Conclusion

**FIGURE 8:**
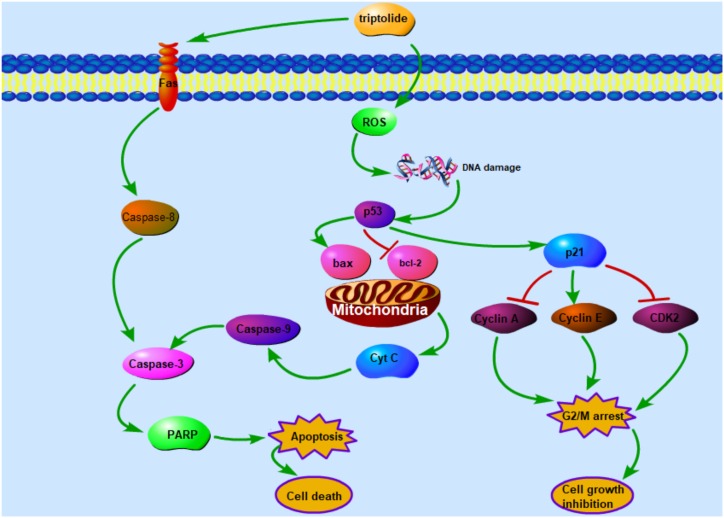
The schematic mechanism of triptolide signaling pathway that inhibits cell growth and leads to cell death in HepaRG cells.

The results of this study reveal that triptolide induces apoptosis in HepaRG cells and inhibits their proliferation through the extrinsic and the intrinsic apoptotic pathways (**Figure 8**). The present study provides important insights into the mechanisms involved in the hepatotoxicity of triptolide.

## Author Contributions

LY, XD, and JN designed the research. BN, JF, CY, XY, and XL performed the experiments. LY, XD, and JN conducted the data analysis. All authors have reviewed and approved the final version of the manuscript.

## Conflict of Interest Statement

The authors declare that the research was conducted in the absence of any commercial or financial relationships that could be construed as a potential conflict of interest.
